# Simple Criteria, Yet the Dearth Utilization-Antithrombotic Management Practice among Atrial Fibrillation Patients at Hawassa University Comprehensive Specialized Hospital, Hawassa, Sidama, Ethiopia

**DOI:** 10.1155/2024/6665787

**Published:** 2024-05-28

**Authors:** Mubarak Hussen, Kindie Woubshet, Seifu Bacha, Worku Ketema

**Affiliations:** ^1^Department of Internal Medicine, College of Medicine and Health Sciences, School of Medicine, P.O. Box 1560, Hawassa, Ethiopia; ^2^New York Internal Medicine Specialty Clinic, Hawassa, Ethiopia; ^3^Panacea Primary Hospital, Hawassa, Ethiopia; ^4^Department of Internal Medicine, Saint Paul Hospital Millennium Medical College, Addis Ababa, Ethiopia; ^5^Department of Pediatrics, College of Medicine and Health Sciences, School of Medicine, P.O. Box 1560, Hawassa, Ethiopia

## Abstract

**Background:**

Atrial fibrillation (AF) is associated with significant mortality and morbidity from stroke and thromboembolism. Despite the availability of effective oral anticoagulation medication, AF patients remain at a high risk of stroke if not treated properly. The purpose of this study was to evaluate antithrombotic therapy practices in patients with AF in the adult cardiac clinic at Hawassa University Comprehensive Specialized Hospital (HUCSH).

**Methods:**

It was a retrospective document review study. Total charts of 119 patients who had follow-up at the adult cardiac clinic with a history of documented AF from January 1 to December 30, 2018, were included. Indicators for antithrombotic therapy based on the congestive heart failure, hypertension, age ≥75 (doubled), diabetes, stroke (doubled), vascular disease, age 65 to 74, and sex category (female) (CHA2DS2-VASc) score were recorded. A *p* value of 0.05 was considered statistically significant. Data analysis was done using SPSS 23 software.

**Results:**

In this study, about 55% of patients with AF were receiving the appropriate antithrombotic treatment. The patients were 48 ± 18.2 years old. Of these, 70% were women. The most frequent underlying cardiac etiology was chronic rheumatic valvular heart disease (50%), followed by cardiomyopathy (14%). In nonvalvular AF, the mean CHA2DS2VASc score was 4.0 ± 1.07. In valvular AF compared to nonvalvular AF, the need for appropriate antithrombotic therapy was substantially greater (*p* 0.0001). Only 8 (13.6%) of the warfarin-using patients had adequate anticoagulation.

**Conclusion:**

The study's findings in regard to antithrombotic usage and maintenance of appropriate antithrombotics for stroke prevention in our patients revealed a discrepancy between recommendations and practice. Therefore, we demand that patients with AF who meet the criteria utilize antithrombotics properly to prevent stroke. Warfarin-taking patients' subpar optimum anticoagulation has to be addressed. Lastly, we advocate proper CHA2DS2-VASc score utilization for nonvalvular heart disease. A regular INR follow-up is also advised for patients who have started taking warfarin.

## 1. Introduction

Atrial fibrillation (AF) is diagnosed by electrocardiography and is characterized by chaotic, rapid, and irregular atrial activation with loss of atrial contraction and irregular ventricular rhythm [1, 2]. AF, which affects 1–2% of the world's population, is the most common kind of cardiac arrhythmia. Age causes AF prevalence to increase rapidly, from 1% in the 40s to 5–15% in the 80s [1, 3].

The global status report of the World Health Organization estimates that 33.5 million people worldwide had AF in 2012 [4, 5]. AF is anticipated to increase in prevalence as the most common persistent cardiac arrhythmia in the general population. AF can be a coincidence, as found in roughly 30–45% of those who had an electrocardiogram for unrelated reasons. The actual estimates of the AF rise may therefore be greatly overstated [6, 7]. The risk factors for the development of AF are age, hypertension, diabetes mellitus, cardiac disease, and sleep apnea.

Hyperthyroidism, acute alcohol use, or acute diseases such as myocardial infarction or pulmonary embolism are examples of acute triggers for AF [6, 8, 9].

AF can be separated into valvular and nonvalvular forms for therapy. Clinical outcomes are all connected to rapid ventricular rates, a lack of atrial involvement in ventricular filling, and a propensity for thrombus development [6, 9].

A major financial burden on healthcare systems, AF carries a high risk of mortality and morbidity from stroke and thromboembolism. Atrial fibrillation patients with thromboembolic stroke (AF contributed to 20% of all stroke) have a death risk that is 1.5–3 times higher than that of those with sinus rhythm. Management of AF by using antithrombotic drugs based on the set criteria is crucial for preventing stroke [1, 6, 10, 11].

Although it is less prevalent in Africa than in developed nations, AF is expected to become much more widespread over the coming years. In Africa, people with AF are generally younger and more likely to have rheumatic valvular heart disease. People with AF have a high mortality rate in Africa [4, 12].

Ethiopia is one of the low-income countries plagued by diseases with a double burden (suffering from both communicable and noncommunicable diseases).

According to the WHO's 2016 report, NCDs were responsible for 38% of all deaths in Ethiopia, with CVDs accounting for 15% of those fatalities [4, 5]. Despite the paucity of information regarding the frequency of atrial fibrillation (AF) in Ethiopia, rheumatic heart disease was determined to be the most common cause of AF (66.3%), with embolic events occurring in approximately 19% of these patients [10, 11, 13]. The World Health Organization has decided to use a group of highly affordable and widely accessible noncommunicable disease (NCD) medicines in order to meet all nine targets by 2025. The ninth target is an 80% availability of the fundamental technologies and essential medications, including generics, needed to treat the main noncommunicable diseases in both public and private facilities, depending on national circumstances. The first global target is a 25% relative decrease in overall cardiovascular disease mortality [4].

International recommendations give simple criteria for the use of antithrombotics in patients with AF; however, it is unknown how frequently these guidelines are really followed in our study area. Besides, to the best of our knowledge, there are no published data regarding the use of antithrombotics in patients with AF in our study area. And hence, this study was aimed at assessing the use of antithrombotics in patients with atrial fibrillation at the adult cardiac clinic of HUCSH, Hawassa, Sidama, Ethiopia.

## 2. Methodology

### 2.1. Study Design and Setting

A retrospective institutional chart review was conducted to evaluate the antithrombotic management practices among cardiac patients with AF at the adult cardiac clinic at HUCSH.

The research site was Hawassa City, which is located in the Sidama National Regional State on the shores of Lake Hawassa in the Great Rift Valley, 273 kilometers south of Ethiopia's capital, Addis Ababa. Cardiac clinic, neurology clinic, endocrinology clinic, and hematology clinic are all available at medical referral clinic (MRC). Over 1200 patients are served by the Adult Cardiac Clinic, which provides follow-up treatments for chronic conditions, including cardiac cases with AF. Cardiac patients with AF are visited on Thursdays every 2 weeks to 4 months. Medical records of AF patients over the last 1 year (January 1 to December 30, 2018 G.C.) were reviewed during the study period of February 29 to March 30, 2019, G.C.

### 2.2. Sample Size Determination and Sampling Technique

The study population included all medical records of patients who were diagnosed with AF and have follow-up at a cardiac clinic between January 1 and December 30, 2018. The charts of cardiac patients who had follow-up were collected from the registry book, and there were about 1200 registered cardiac patients, of which 140 were documented AF patients, and all AF patients were chosen as study participants.

### 2.3. Source Population

The source population included all cardiac patients who were followed up at HUCSH's adult cardiac referral clinic.

### 2.4. Study Population

The study population included all cardiac patients who had been diagnosed with AF.

### 2.5. Inclusion and Exclusion Criteria

#### 2.5.1. Inclusion Criteria

Patients over the age of 18 with documented cardiac disease and AF who were being followed up on at HUCSH's adult cardiac referral clinic.

#### 2.5.2. Exclusion Criteria

Patients who have had discontinued a follow-up, patients on anticoagulation for another reasons other than AF, and patients enrolled in a clinical trial were excluded from the study.

### 2.6. Operational Definitions

Adherence to standard protocol: guideline adherence was based on compliance with antithrombotic recommendations for AF stroke prevention according to the CHA2DS2-VASc score [1, 2].

Valvular AF: AF that occurs in association with a prosthetic heart valve, valve repair, or rheumatic valvular heart diseases.

Nonvalvular AF: AF in the absence of rheumatic mitral stenosis, a mechanical or bioprosthetic heart valve, or mitral valve repair.

Labile International Normalized Ratio (INR): unstable/high INR and time in therapeutic range <60%.

Appropriate therapy: utilization of oral anticoagulation for CHA2DS2-VASc scores of 2 or above or for patients with mitral valve disease, and no therapy or antiplatelet therapy, or an oral anticoagulation (OAC) might have been considered for CHA2DS2-VASc scores of 1 or lower, therapy could be forgone for CHA2DS2-VASc scores of zero.

Inappropriate therapy: no anticoagulation therapy with a CHA2DS2-VASc score of 2 and above or any anticoagulation therapy with a CHA2DS2-VASc score of zero [2, 7].

Optimal anticoagulation: a patient on warfarin whose INR value is in the therapeutic range of 2 to 3 basically 60% of the time [2, 7].

Monotherapy: a single class of antithrombotic medication used.

Dual therapy: two different classes of antithrombotic medication used [2, 7].

### 2.7. Variables

#### 2.7.1. Dependent Variables

The practice of antithrombotic utilization among AF patients based on set criteria was the dependent variable.

#### 2.7.2. Independent Variables

Age, sex, type of underlying cardiac disease, type of AF, and number of antithrombotics were the independent variables in this research.

### 2.8. Data Collection

Prior to data collection, two general practitioners (GPs) were hired and given a day of training on how to gather the necessary data from log books and patient charts using organized and tested questionnaires. All of the follow-up charts for cardiac patients were collected, and all of the charts with AF noted were taken. The lead investigator, together with the supervisor, was checking for the completeness of the collected data.

### 2.9. Data Quality Assurance

The primary investigator oversaw the data gathering procedure, and daily checks were made to ensure that the data were accurate and complete. One week before the actual data collection, the investigator did a pretest on the patient charts to ensure consistency, accuracy, and to correct any ambiguity in the questionnaires before the actual data were collected.

### 2.10. Data Processing and Analysis

The consistency and completeness of the collected data were checked. The data were analyzed using SPSS version 23 software after all the data had been entered. Standard deviations (SD), percentages, and means were used to report the results. The crude odds ratio (OR) and 95% confidence interval (CI) for each relevant variable were calculated. Finally, adjusted odds ratios were calculated using bivariate and multivariate logistic regression, and *p*=0.25 was chosen as a meaningful threshold for selecting candidate variables for multiple regression, with *p* value less than 0.05 regarded statistically significant.

## 3. Results

### 3.1. Sociodemographic Characteristics of Study Participants

There were 1200 patients at the cardiac clinic, of which 700 (58) were females. Out of these, 140 patients had AF with 119 meeting the inclusion criteria. The most common reason for exclusion was another indication for anticoagulation, predominantly for trial purposes. The prevalence of AF was 11.6% in the cardiology clinic. Most patients are from the Oromia region (54.6%) and about 18% are from the Hawassa city. Of a total of 119 patients who had AF, 83 (70%) were females and 36 (and 30%) were males. The mean age of the patients is 48 ± 18.2 with an age range of 18–85 years old and a median age of 50. Males are older than females 53.8 vs. 46.1 (*p* < 0.05). The majority of patients had underlying chronic rheumatic valvular heart disease (CRHD) (50%), followed by cardiomyopathy (14%), ischemic heart disease (13%), and hypertension (12%). About 32 (26.9%) patients have associated comorbidities, stroke accounts for 17 (14.3%), and diabetes mellitus was present in 10 (8.4%) patients and chronic kidney disease 3 (2.5) patients (Table 1).

### 3.2. Clinical Characteristics of Study Participants

Out of 119 individuals in our research population, 113 (94.9%) had an indication for getting antithrombotic, 4 (3.4%) had an optional antithrombotic, and 2 (1.6%) did not have an indication for antithrombotic according to the guidelines. Of the 113 patients with an indication for OAC, 49 (43.3%) received treatment, while 64 (56.7%) did not, despite being fulfilling the criteria. Three of the four patients in whom antithrombotic therapy is optional received OAC, while one of the two patients who had no rationale for OAC received OA. Echocardiography was performed for all patients. The majority of patients with persistent rheumatic valvular heart disease have mitral stenosis (77%). Females had a larger prevalence of CRHD than males (81% vs. 19%, *p*=0.008).

The majority of patients received a score of 2 or 3, accounting for 62.5% of all nonvalvular AF patients. The average CHADS2VASc score was 4.0 ± 1.07. The mean HAS-BLED score was 1.56 ± 0.64, and all patients had a score of less than 3 if alcohol history was excluded. Nonvalvular AF patients (93%) had CHADS2VASc scores ≥2, emphasizing the necessity for oral anticoagulants as the recommended method for stroke prevention. Overall, 25 (21%) of our patients were given antiplatelet (aspirin), 53 (44.5%) were given oral anticoagulants (OAC), and 6 (5% were given both OAC and antiplatelets).

Significantly, 35 patients (29.5%) did not receive any type of stroke prevention therapy, including aspirin or OAC. At the age categories of 65, 65–74, and 75 years, the distribution of patients on suitable antithrombotic care was 60%, 29.2%, and 50%, respectively (Table 2).

About 65 (55.6%) patients with AF received the proper antithrombotic treatment at the cardiac clinic. However, 54 (45.4%) of the AF patients received ineffective care. Contrary to nonvalvular AF, where only 20 (33.3%) of cases are effectively managed, the majority of valvular AF (46 (88%)) is treated with the proper antithrombotic medication.

Only a tiny portion of warfarin-taking patients, 8 patients or 13.3%, had INRs in the therapeutic range of 2.0 to 3. The majority of patients, 52 patients or 86.7%, are not on optimum anticoagulation.

For the past three months' worth of monthly INR readings, 33%, 37%, and 39% are below therapeutic levels. 13%, 10%, and 6% are overanticoagulated, while 17%, 13%, and 11% are ideal.

Warfarin is prescribed for 87% of patients with underlying chronic rheumatic heart disease, and aspirin is prescribed for 13% of these individuals. 85% of patients with NVAF had a CHA2DS2-VASc score of 2 to 5. Aspirin was the antithrombotic drug of choice in 20 (47%) of the nonvalvular AF patients; warfarin was used alone in 16 (38%) of the patients; and a combination of aspirin and warfarin was used in 6 (14%) of the patients with a CHA2DS2-VASc score >2 (Table 3).

Table 3 shows the antithrombotic treatment according to the CHAD2S2-VAS score for AF among adult cardiac patients at HUCSH, Sidama, Ethiopia, from February 29 to March 30, 2019.

About 20 (33.3%) patients received the proper antithrombotic treatment for nonvalvular AF in the cardiac follow-up clinic.

However, 40 (66.6%) NVAF patients were improperly handled, and 95.2% of them had a CHA2DS2-VASc score of less than 2 (Table 3).

### 3.3. Factors Associated with Antithrombotics Management Practice

After bivariate analysis, cumulative odds ratio (COR) found a statistically significant correlation between the different forms of AF and the appropriate treatment. Additionally, individuals who had received more prescriptions for drugs were more likely to receive proper antithrombotic therapy (COR = 3.717, 95% confidence interval [CI (1.07–12.87)]) (Table 4).

Using bivariate and multivariate logistic regression techniques, the influence of specific sociodemographic, clinical, and other factors on the appropriateness of an antithrombotic management strategy was examined. As a result, the following factors were taken into account in the bivariate analysis: age category, sex, residency, AF type, presence of comorbidities, and number of medicines. The multiple logistic regressions comprised explanatory variables with *p* values up to 0.25. The optimal antithrombotic management practice is substantially correlated with the type of AF, age, and quantity of medications. According to multivariate analysis, those with valvular AF (AOR = 10.5; 95% CI: 3.17–34.61) had a higher likelihood of receiving the right treatment than patients without valvular AF. Patients on dual antithrombotic medications are more likely to use effective management techniques (AOR = 6.025; 95% CI = 1.84–19.71). Other factors like age, residence (urban vs. rural), sex, the number of drugs taken, the CHA2DVASC score, and the hypertension, abnormal liver/renal function, stroke history, bleeding history or predisposition, Labile INR, elderly, and drug/alcohol usage (HAS BLED) score were not substantially linked to how appropriate the antithrombotic therapy was (Table 5).

## 4. Discussion

The American College of Cardiology (ACC), American Heart Association (AHA), and European Society of Cardiology (ESC) recommend antithrombotic therapy based on a patient's thromboembolic risk. They advocate choosing the right antithrombotic therapy for patients with nonvalvular AF using the CHA2DS2-VASc score approach. It is advised to forgo antithrombotic therapy because the maximum CHA2DS2-VASc score is 9, and a score of 0 indicates a “really low risk.” The ESC guidelines on AF recommend or prefer oral anticoagulation for patients with one or more stroke risk factors (i.e., a CHA2DS2-VASc score of 1.0 and above). For people with valvular AF, warfarin antithrombotic treatment with a target INR of 2.0–3.0 is indicated. Despite these well-publicized findings, many patients with atrial fibrillation get insufficient thromboprophylaxis [1, 2, 14–16]. In this study, 70% of the patients with AF were female, in contrast to earlier investigations where the prevalence of AF was higher in males [7]. The most prevalent cardiac problems in this study, CRVHD, are typical in females and might be the cause for the observed disparity.

Our patients were 48 + 18.2 years old on average. The average age of males was substantially greater than that of females (53.8 vs. 46.1, *p*=0.037). In this study, the average age of AF patients was lower than the average age of AF patients in other studies, which ranged from 60 to 70 years [7, 17, 18].

Chronic rheumatic valvular heart disease (CRHD) was the most common condition among AF patients, followed by cardiomyopathy (14%), ischemic heart disease (13%), and hypertension (12%). Similar findings came from a study conducted in India, where 51.5% of patients had RHD [7, 17]. In this study, 65 (55.6%) patients with AF received the proper antithrombotic therapy at the cardiac clinic. However, 54 (45.4%) individuals with AF received suboptimal care. Of the 59 (49.5%) patients with valvular AF who had a clear indication for anticoagulation therapy with warfarin, 47 (88%) were given the proper care, with the remaining 12% receiving improper management. About 54 (90%) of the patients with nonvalvular AF had a CHA2DS2 VASc score of 2.0 or higher, necessitating anticoagulation; however, only 34.4% of these patients were given warfarin in this study. Antiplatelet treatment, specifically aspirin, was more frequently administered to patients with ischemic heart disease. In comparison to other nations like India, where the rate of properly reported antithrombotic management ranged from 70% to 90%, the NVAF has a poor rate of antithrombotic management [10, 11, 18, 19].

We were able to show that the rate of adequate antithrombotic management, which ranged from 38 to 55%, was comparable to previously reported rates in other countries despite the study's relatively small sample size [7, 14, 20, 21].

This study shows that the use of antithrombotics is unaffected by the patient's age.

The rates of proper antithrombotic usage in these people were 60%, 29%, and 50%, respectively, in contrast to a study from Cameroon, where the rate increased from 72% in the group of patients aged 65 and under to 87% in the group of patients aged >75 years [7].

Additionally, we found that valvular AF substantially used optimal antithrombotic than nonvalvular AF (*p*=0.0001). This may be a result of the treating physicians' related effect on stratifying their patients for CHA2DS2-VASc score. Maintaining the standard of anticoagulation control has a significant impact on the effectiveness and dangers of oral anticoagulation. Warfarin has a narrow therapeutic range of 2.0–3.0, which makes it challenging to dose and monitor but can dramatically lower the risk of stroke. Only a tiny portion of patients (13.3%) had an INR in the therapeutic range of 2.0–3.0 among this study's warfarin-using patients; the remainder (86.7%) is not receiving optimal anticoagulation. The status of anticoagulant therapy among AF patients has been assessed by numerous sizable randomized trials. These rates are greater than what we found in our analysis; in several randomized trials, 60–69% of patients had INRs that were in the therapeutic range. Despite the fact that participants in these studies' randomized trials received a lot of care and attentive monitoring, 60–69% of them showed signs of having adequate anticoagulation. The different causes of inadequate anticoagulation in our patients may include inadequate patient education, problem with patient adherence, and drug accessibility [3, 7, 10, 11, 22].

### 4.1. Limitations and Strengths of the Study

This study has some drawbacks, such as the fact that it is a retrospective document review, which will make it difficult to follow the progress of the participants. In order to determine the therapeutic significance of following the guideline, it can be challenging to obtain the chronology of the comorbidities versus AF. Besides, there is just one institution involved in this retrospective evaluation of medical records, and the sample size is small, endangering the reliability and quality of the findings. It was challenging to examine the physician profile with regard to the appropriate use of antithrombotics among AF patients because the study was conducted in one of the nation's teaching hospitals, where the turnover of doctors, medical residents, and medical interns is quite frequent.

The study's strength lies being conducted in one of the tertiary hospitals in the country, an ideal setting for the provision of optimal care. As such, the results will contribute to better quality care.

## 5. Conclusion

The study's findings in regard to antithrombotic usage and maintenance of appropriate antithrombotics for stroke prevention in our patients revealed a discrepancy between recommendations and practices. Therefore, we demand that patients with AF who meet the criteria utilize antithrombotics properly to prevent stroke. Warfarin-taking patients' subpar optimum anticoagulation has to be addressed. Lastly, we advocate proper CHA2DS2-VASc score utilization for nonvalvular heart disease. A regular INR follow-up is also advised for patients who have started taking warfarin.

## Figures and Tables

**Table 1:** Sociodemographic characteristics of study participants among adult cardiac patients at HUCSH, Sidama, Ethiopia, from February 29 to March 30, 2019.

Variables	Frequency	Number
Age in years	48 (18–85)	119
Below 65	71.4%	85
66–74	20.2%	24
≥75	8.4%	10
Sex (female)	70%	86
Residence		
Urban	36.1%	43
Rural	63.9%	76
Types of AF		
Valvular	49.6%	59
Nonvalvular	50.4%	60
Underlying cardiac diseases		
CRVHD	50%	59
IHD	12%	14
HHD	13%	15
Cardiomyopathy	14%	17
Thyrocardiac	2.5%	3
DVHD	5%	6
Cor pulmonale	2.5%	3
CHA2DS2–VASc score^£^		
Score = 0	3.4	2
Score = 1	6.6%	4
Score ≥2	90%	54
Comorbid illness, (yes)	26.8%	32
Antithrombotic		
Monotherapy	65.5%	78
Dual therapy	5%	6
None	29.5%	35
Appropriateness of AF management		
Appropriate	55%	65
Inappropriate	45%	54

^£^Congestive heart failure, hypertension, age ≥75 (doubled), diabetes, stroke (doubled), vascular disease, age 65 to 74, and sex category (female).

**Table 2:** Relationship of sociodemographic characteristics with AF among adult cardiac patients at HUCSH, Sidama, Ethiopia, from February 29 to March 30, 2019.

Characteristics	Appropriate (%)	Inappropriate (%)	*X* ^2^ test	*p* value
Age (mean)	43 ± 17.2	54 ± 18.7	9.86	0.007
Below 65	51 (60%)	34 (40%)		
66–74	7 (29.2%)	17 (79.8%)		
≥75	5 (50%)	5 (50%)		
Sex			3.495	0.062
Male	15 (41.7%)	21 (58.3%)		
Female	48 (57.8%)	35 (42.2%)		
Residency			6.231	0.013
Urban	30 (46.2%)	13 (24.1%)		
Rural	35 (53.8%)	41 (75.9%)		
Types of AF			22.902	0.000
Valvular	46 (88%)	13 (22)		
Nonvalvular	20 (33.3%)	40 (66.7%)		
Antithrombotic drug the patient taking			19.961	0.000
Monotherapy	51 (78.5%)	29 (53.7%)		
Dual therapy	6 (9.2%)	0 (0%)		
None	8 (12%)	25 (46.3%)		
Comorbid illness?			2.385	0.122
Yes	14 (22.2%)	18 (35.3%)		
No	49 (77.8%)	33 (64.7%)		
Has bled score^£^			3.222	0.359
0	13 (46.4%)	24 (55.8%)		
1	12 (42.9%)	16 (37.2%)		
2	3 (10.7%)	3 (7.0%)		

^£^Hypertension, abnormal liver/renal function, stroke history, bleeding history or predisposition, labile INR, elderly, drug/alcohol.

**Table 3:** Antithrombotic treatment according to CHA2DS2–VAS score in Hawassa University comprehensive specialized hospital, Southern Ethiopia, February 30 to March 29, 2019.

CHADVASC*∗*score	Aspirin	Warfarin	Combination of aspirin and warfarin	Total
0	0 (0%)	1 (6.25%)	0 (0%)	1 (2.3%)
1	0 (0%)	3 (18.7%)	0 (0%)	3 (7.1%)
2	7 (35.0%)	1 (6.25%)	0 (0%)	8 (19.0%)
3	5 (25.0%)	5 (31.25%)	4 (66.7%)	14 (33.3%)
4	5 (25.0%)	6 (37.5%)	2 (33.3%)	13 (30.9)
5	1 (5.0%)	0 (0%)	0 (0%)	1 (2.3%)
6	2 (10.0%)	0 (0%)	0 (0%)	2 (4.7%)
Total	20 (100.0%)	16 (100.0%)	6 (100.0%)	42 (100.0%)

^*∗*^Congestive heart failure, hypertension, age, diabetes mellitus, vascular disease, stroke, and sex.

**Table 4:** Bivariate analysis of risk factor variables for AF among adult cardiac patients at HUCSH, Sidama, Ethiopia, from February 29 to March 30, 2019.

Characteristics	Receiving proper anticoagulant therapy (%)	Not receiving proper anticoagulant therapy (%)	COR ˜ (95% CI)	*p* value
Age (mean)	43 ± 17.2	54 ± 18.7		
Below 65	51 (60%)	34 (40%)	Ref.	
65–74	7 (29.2%)	17 (79.8%)	4.23 (1.58–11)	0.004
≥75	5 (50%)	5 (50%)	3.3 (0.65–19.8)	0.160
Sex				
Male	15 (41.7%)	21 (58.3%)	2.73 (0.96–7.8)	0.064
Female	48 (57.8%)	35 (42.2%)	Ref.	
Residence				
Urban	30 (46.2%)	13 (24.1%)	0.370 (0.17–0.82)	0.014
Rural	35 (53.8%)	41 (75.9%)	Ref.	
Type of AF				
Valvular AF	46 (88%)	13 (12)	6.84 (3.01–15.56)	0.000
Nonvalvular AF	20 (33.3%)	40 (66.7%)	Ref.	
Antithrombotic drug				
Monotherapy	51 (78.5%)	29 (53.7%)	1.50 (1.0–2.21)	0.001
Dual therapy	6 (9.2%)	0 (0%)	3.72 (1.1–12.9)	0.000
None	8 (12%)	25 (46.3%)	0.000	000
Comorbid illness?				
Yes	14 (22.2%)	18 (35.3%)	1.26 (0.46–3.4)	0.125
No	49 (77.8%)	33 (64.7%)	Ref.	
HAS BLED score^€^				
0	13 (46.4%)	24 (55.8%)	3.54 (0.57–22.03)	0.263
1	12 (42.9%)	16 (37.2%)	3.1 (0.48–19.84)	0.157
2	3 (10.7%)	3 (7.0%)	Ref.	

^€^Hypertension, abnormal liver/renal function, stroke history, bleeding history or predisposition, labile INR, elderly, drug/alcohol. ˜Cumulative odds ratio. *p* value <0.25 was taken as the candidate variable for multivariate analysis.

**Table 5:** Factors associated with AF management among adult cardiac patients at HUCSH, Sidama, Ethiopia, from February 29 to March 30, 2019.

Characteristics	Appropriate (%)	Inappropriate (%)	COR (95% CI)	AOR (95% CI)
Age (mean)	43 ± 17.2	54 ± 18.7		
Below 65	51 (60%)	34 (40%)	Ref.	
65–74	7 (29.2%)	17 (79.8%)	4.23 (1.58–11)	1.47 (0.25–8.8)
≥75	5 (50%)	5 (50%)	3.26 (0.65–19.8)	(0.38–15.5)
Sex				
Male	15 (41.7%)	21 (58.3%)	2.73 (0.96–7.8)	1.05 (0.35–3.2)
Female	48 (57.8%)	35 (42.2%)	Ref.	
Residence				
Urban	30 (46.2%)	13 (24.1%)	0.370 (0.17–0.82)	0.34 (0.12–0.99)
Rural	35 (53.8%)	41 (75.9%)	Ref.	
Type of AF				
Valvular AF	46 (88%)	13 (12)	6.84 (3.1–15.56)	10.5 (3.2–34.6)
Nonvalvular AF	20 (33.3%)	40 (66.7%)	Ref.	
Antithrombotic drug				
Monotherapy	51 (78.5%)	29 (53.7%)	1.50 (1.02–2.21)	Ref.
Dual therapy	6 (9.2%)	0 (0%)	3.72 (1.1–12.9)	6.03 (1.8–19.7)
None	8 (12%)	25 (46.3%)	0.000	000
Comorbid illness?				
Yes	14 (22.2%)	18 (35.3%)	1.26 (0.46–3.4)	0.67 (0.2–2.3)
No	49 (77.8%)	33 (64.7%)	Ref.	

## Data Availability

All relevant data are available within the manuscript.
